# Respiratory complex I in mitochondrial membrane catalyzes oversized ubiquinones

**DOI:** 10.1016/j.jbc.2023.105001

**Published:** 2023-06-30

**Authors:** Ryo Ikunishi, Ryohei Otani, Takahiro Masuya, Kyoko Shinzawa-Itoh, Tomoo Shiba, Masatoshi Murai, Hideto Miyoshi

**Affiliations:** 1 Division of Applied Life Sciences, Graduate School of Agriculture, Kyoto University, Kyoto, Japan; 2 Department of Life Science, Graduate School of Life Science, University of Hyogo, Hyogo, Japan; 3 Department of Applied Biology, Graduate School of Science and Technology, Kyoto Institute of Technology, Kyoto, Japan

**Keywords:** bioenergetics, respiratory enzymes, complex I, ubiquinone, chemical biology

## Abstract

NADH-ubiquinone (UQ) oxidoreductase (complex I) couples electron transfer from NADH to UQ with proton translocation in its membrane part. The UQ reduction step is key to triggering proton translocation. Structural studies have identified a long, narrow, tunnel-like cavity within complex I, through which UQ may access a deep reaction site. To elucidate the physiological relevance of this UQ-accessing tunnel, we previously investigated whether a series of oversized UQs (OS-UQs), whose tail moiety is too large to enter and transit the narrow tunnel, can be catalytically reduced by complex I using the native enzyme in bovine heart submitochondrial particles (SMPs) and the isolated enzyme reconstituted into liposomes. Nevertheless, the physiological relevance remained unclear because some amphiphilic OS-UQs were reduced in SMPs but not in proteoliposomes, and investigation of extremely hydrophobic OS-UQs was not possible in SMPs. To uniformly assess the electron transfer activities of all OS-UQs with the native complex I, here we present a new assay system using SMPs, which were fused with liposomes incorporating OS-UQ and supplemented with a parasitic quinol oxidase to recycle reduced OS-UQ. In this system, all OS-UQs tested were reduced by the native enzyme, and the reduction was coupled with proton translocation. This finding does not support the canonical tunnel model. We propose that the UQ reaction cavity is flexibly open in the native enzyme to allow OS-UQs to access the reaction site, but their access is obstructed in the isolated enzyme as the cavity is altered by detergent-solubilizing from the mitochondrial membrane.

Proton-translocating NADH-ubiquinone (UQ) oxidoreductase (complex I) couples electron transfer from NADH to UQ with the translocation of protons across the membrane, generating the proton motive force required for ATP synthesis and substrate transport across the membrane (1, 2, 3, 4). Complex I is the largest (∼1 MDa) respiratory chain enzyme and one of the major sources of superoxide production in mammalian mitochondria (4). Recent progress in single-particle cryo-EM (5, 6, 7, 8, 9, 10, 11, 12, 13, 14, 15, 16, 17) and computational simulation studies (18, 19, 20, 21, 22) has provided valuable information about the structure and functions of the enzyme. The consensus view has been that structural and electrostatic rearrangements induced by UQ reduction transmit to the membrane subunits to trigger proton translocation. Therefore, UQ reduction plays a key role in energy conversion processes, although the mechanism responsible has not been fully elucidated.

Structural biology studies have identified a long and narrow tunnel-like cavity (∼30 Å long), which extends from a narrow entrance (∼5 Å diameter) located in the middle of the membrane-embedded subunit ND1 to the Fe-S cluster N2 (Fig. S1). It is widely accepted that UQs possessing different isoprenyl chain lengths enter and transit this UQ-accessing tunnel to be reduced at the “top” of the tunnel near cluster N2 and then move backward, exiting into the membrane (5, 6, 7, 8, 9, 10, 11, 12, 13, 14, 15, 16, 17). The so-called quinone-site inhibitors of a variety of chemical frameworks are considered to block the catalytic reaction of UQ by occupying the UQ-accessing tunnel (11, 14, 17, 23, 24), although the binding positions in the tunnel vary depending on their chemical properties. In contrast to this canonical model for the UQ reaction, a “two-UQ” model has also been proposed (15, 25, 26): a single UQ_10_ molecule is reduced at the distal position near the cluster N2 and moves to the proximal position near the entrance, but does not exit from the tunnel. Instead, this reduced UQ_10_ transfers electrons to a secondary (exchangeable) UQ_10_ that may be bound outside the tunnel, and, then, the formed oxidized UQ_10_ in the tunnel returns to the distal position for the next catalytic cycle. Nevertheless, the binding position of the secondary UQ_10_ has yet to be identified.

Chemical biology studies conducted in our laboratory demonstrated that the binding of a variety of ligand molecules (substrate UQs, inhibitors, and regulators) to complex I cannot be explained simply by the scenario that all ligand molecules enter the canonical UQ-accessing tunnel (27, 28, 29, 30, 31, 32). Of particular interest is the catalytic reaction of oversized UQs (OS-UQs) in the enzyme (29). OS-UQs have an extremely bulky and rigid block (1-methoxy-2,6-di(3-methoxy-3-methyl-1-butynyl)benzene), attached to the end of their side chains of varying lengths (Figs. 1 and S2), to prevent the UQ head-ring from accessing the reaction site located deep in the tunnel. The diameter of this block (∼13 Å across) is much wider than the diameter of the tunnel of bovine complex I (the entry point is ∼3 × 5 Å, ref. 5). Molecular dynamics simulations based on a cryo-EM structure of bovine complex I (5) indicated that the block is too large to enter and transit the narrow UQ tunnel (29). Therefore, if the side chain is not long enough, the UQ head ring is unable to reach its catalytic site at the end of the tunnel nearby cluster N2. However, in an assay using bovine heart submitochondrial particles (SMPs), amphiphilic OS-UQ2 and OS-UQ3 were able to function as electron acceptors from the native complex I (29). The reduction of OS-UQ2 and OS-UQ3 and proton-translocation coupled with the reduction were fully inhibitor-sensitive, indicating that the reaction of these OS-UQs takes place at the physiological catalytic site of the enzyme. This finding is difficult to reconcile with the UQ-accessing tunnel model.

To further explore the reaction of OS-UQs, we also investigated analogs possessing much longer isoprenyl side chains (OS-UQ6–OS-UQ8, Fig. 1). As these OS-UQs are extremely hydrophobic (their solubilities in water are very low), their electron transfer activities could not be determined by directly adding to a suspension of SMPs, as conducted for the amphiphilic OS-UQ2 and OS-UQ3 above. In this regard, Hirst and colleagues (33) established an excellent proteoliposome system to measure the electron transfer activity of extremely hydrophobic UQs, such as UQ_10_, in the catalytic reaction mediated by complex I. Their proteoliposomes contain the isolated bovine complex I, alternative quinol oxidase (AOX) isolated from *Trypanosoma brucei*, and UQ of choice. The electron flow in the proteoliposomes is schematically shown in Fig. S3*A*. AOX is a cyanide-insensitive single-subunit quinol oxidase and catalyzes UQH_2_ oxidation and O_2_ reduction without proton translocation. AOX was reconstituted into or externally added to the proteoliposomes to re-oxidize UQH_2_ to UQ (24, 33). Because of the marked hydrophobicities of OS-UQ6–OS-UQ8, we made use of this proteoliposome system to examine their electron transfer activities.

**Figure 1:**
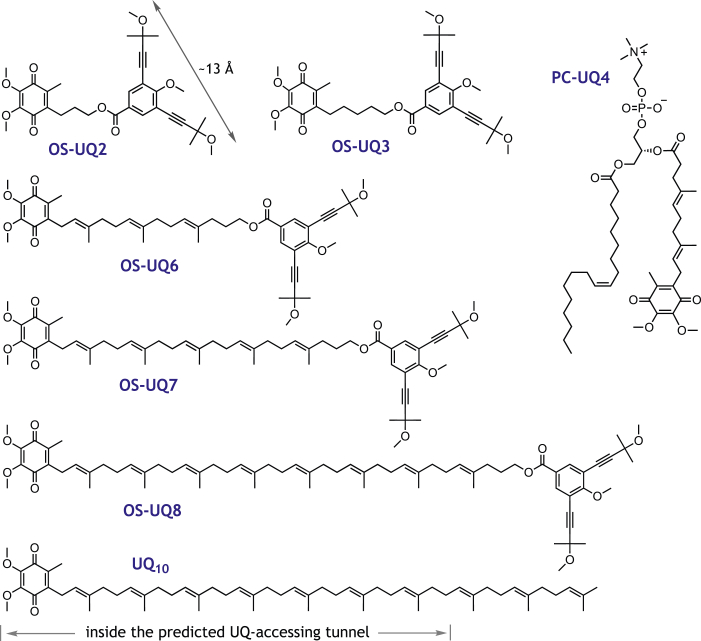
**Structures of oversized ubiquinones (OS-UQs) and PC-UQ4 studied in this study.** The structure of UQ_10_ is also shown as a reference. Based on the modeled structure of bovine complex I with bound UQ_10_ (*16*), the side chain moiety located inside the predicted UQ-accessing tunnel is indicated.

Contrary to our expectation, OS-UQ6 and OS-UQ7 could not be catalytically reduced by the isolated complex I in the proteoliposomes, whereas OS-UQ8, which is predicted by the structure to be just long enough (Fig. 1), could be reduced as efficiently as UQ_10_ (29). The electron transfer activity of OS-UQ2 and OS-UQ3 could not be evaluated in the proteoliposomes because these OS-UQs could not function as substrates of AOX. Alternatively, by HPLC analysis of the UQ content extracted from the proteoliposomes, we found that OS-UQ2 and OS-UQ3 cannot be reduced by the isolated complex I (see ref. 29 for details). Thus, contrary to the results obtained in SMPs, the results in the proteoliposomes can be viewed as evidence for the UQ tunnel model. Altogether, as OS-UQs exhibited different reaction behaviors between the native membrane system and proteoliposomes, no definite conclusion could be drawn on the physiological relevance of the canonical UQ tunnel in the previous study (29). Later, by producing pairs of wide and narrow UQs (pUQ_*m*_ vs. pUQ_*p*_, Fig. S2), with each pair having the same physicochemical properties except for a slight difference (∼1 Å) in the side chain widths, we demonstrated that the different reaction behaviors of OS-UQs between the two enzymes are not peculiar to these bulky UQs; rather, it is also the case for much less bulky UQs (32).

To explain the different reaction behaviors of UQs between the native and isolated complex I, we had tentatively hypothesized that the flexible UQ reaction cavity in the native enzyme is altered by detergent-solubilizing from the inner mitochondrial membrane (29, 32); therefore, steric obstruction, which restricts the access of UQ to the deep reaction site, may be different between the two enzymes. As is sometimes the case with membrane-bound proteins, an inherent question is to what extent detergent-solubilized proteins maintain all properties of the proteins (34, 35, 36, 37, 38, 39). Complex I would be no exception. In fact, a cryo-EM study on mouse complex I with two bounds piericidin A molecules suggested that the properties of the distal section of the UQ tunnel may be somewhat affected by the loss of specific phospholipids that are important for complex I activity and/or the presence of detergent (10).

With this background, unless we can fully rule out the possibility that the UQ reaction cavity is altered by detergent-solubilizing, the reaction of OS-UQs must be uniformly assessed using the native enzyme in the inner mitochondrial membrane. To do this, the assay system using SMPs fused with liposomes that incorporate extremely hydrophobic OS-UQ should be adequate, as demonstrated by Hackenbrock and colleagues (40) to investigate how lateral diffusion of UQ_10_ in UQ_10_-enriched mitoplasts by fusion with liposomes incorporating UQ_10_ affects the overall electron transfer rates (Fig. S3*B*). In addition to this, supplying AOX to the fused SMPs to recycle UQH_2_ to UQ should be experimentally advantageous. Fedor and Hirst (41) showed that externally added AOX catalyzes reoxidation of endogenous UQ_10_H_2_ to UQ_10_ in bovine SMPs in the presence of cyanide to block UQ_10_H_2_ oxidation by complexes III/IV, enabling the UQ_10_-mediated electron transfer between complex I and AOX (Fig. S3*C*).

To elucidate the physiological relevance of the canonical tunnel model, here we investigated the native complex I-catalyzed reduction of OS-UQs using this SMP assay system (abbreviated as “SMPs-Lipo-AOX”, Fig. S3*D*). All OS-UQs tested could be reduced by the native enzyme in the SMPs-Lipo-AOX. The reduction of OS-UQs and proton translocation coupled with the reduction was fully inhibitor sensitive. The present study provides definite experimental data to refute the physiological relevance of the UQ-accessing tunnel model.

## Results

### UQ_10_-mediated electron transfer between complex I and AOX in SMPs

**Figure 2:**
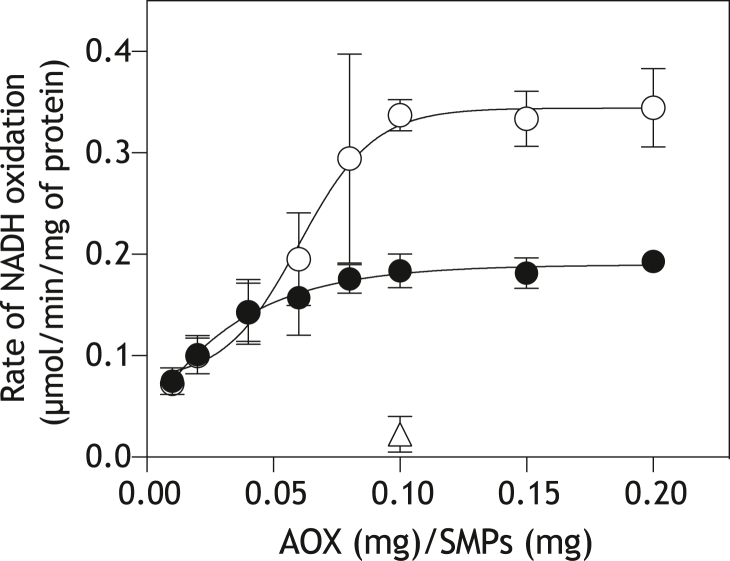
**Effects of AOX on the NADH oxidation in SMPs.** The isolated AOX was added to ordinary SMPs (*open circles*) or SMPs fused with liposomes composed of phospholipids alone (*closed circles*) in the presence of 4.0 mM KCN. The reaction was initiated by adding NADH (100 μM) after incubation of SMPs with AOX for 4 min at 30 °C. The final concentration of SMP protein was set to 30 μg/ml for both assays. The *open triangle* indicate the NADH oxidation rate at 0.10 mg AOX/mg SMP protein in the presence of 1.0 μM ascofuranone. Values are means ± S.E. (*n* = 3).

The NADH oxidation in bovine heart SMPs (30 μg protein/ml) was measured in the presence of varying concentrations of AOX and 4.0 mM cyanide (Fig. 2). AOX efficiently binds to the inner mitochondrial membrane surface (41, 42). As the oxidation of endogenous UQ_10_H_2_ by complexes III/IV is blocked by cyanide, the NADH oxidation proceeds via UQ_10_-mediated electron transfer between complex I and AOX (33). The NADH oxidation rate, which was determined 30 s after the addition of NADH, increased with increasing AOX and reached a plateau at around 0.10 mg AOX/mg SMP protein (*open circles* in Fig. 2). We confirmed that the NADH oxidation is almost completely blocked by ascofuranone (*open triangle*), a specific inhibitor of AOX (42).

AOX titration was also carried out using SMPs that were fused with liposomes, which were composed of phospholipids alone without UQ, in a weight ratio of 1:2 (mg SMP protein/mg liposomal phospholipids), as shown in Figure 2 (*closed circles*). The phospholipids, predominantly containing phosphatidylcholine, phosphatidylethanolamine, and cardiolipin, were extracted from the isolated bovine heart mitochondria in our laboratory (29). A portion of the mixture of SMPs and liposomes, which underwent freeze-thawing treatment (see Experimental Procedures), was directly subjected to the NADH oxidation assay. Although the NADH oxidation rates decreased compared with the non-fused SMPs as the average distance between complex I and AOX increased due to incorporated liposomal phospholipids (40) (described in detail in the next section), the activity increased with increasing AOX and reached a plateau at ∼0.10 mg AOX/mg SMP protein. Based on the results, we set the AOX concentration at 0.10 mg AOX/mg SMP protein in the following experiments. Note that AOX purification requires a detergent *n*-dodecyl-β-D-maltoside (DDM) to solubilize it in solution. Consequently, the addition of increasing amounts of AOX to SMPs also adds increasing amounts of DDM, which may alter the membrane integrity (41). Therefore, we did not use AOX at over 0.10 mg AOX/mg SMP protein.

### Fusion of SMPs with liposomes

**Figure 3:**
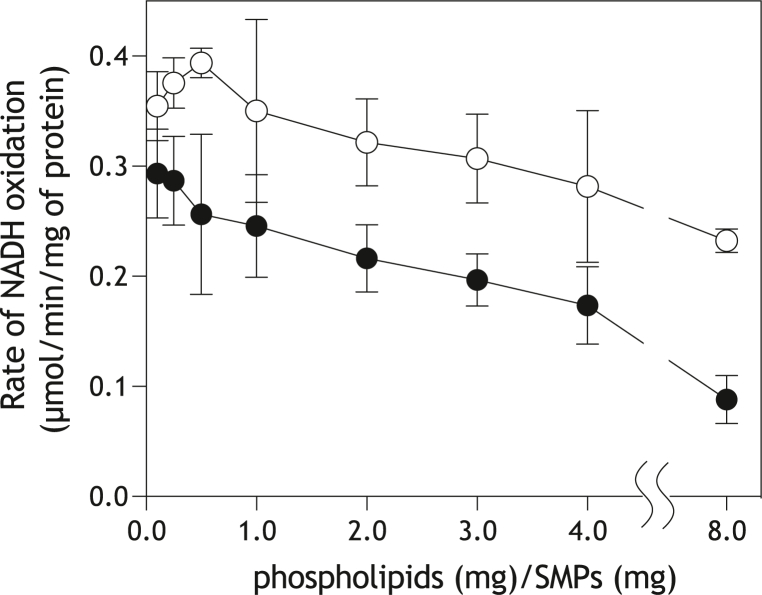
**The NADH oxidation in SMPs fused with liposomes**. The NADH oxidation rate was measured in SMPs, which were fused with liposomes composed of phospholipids alone (*closed circles*) or phospholipids plus 11.8 (±2.7) mM UQ_10_ (*open circles*). The concentrations of AOX and KCN were set to 0.10 mg AOX/mg SMP protein and 4.0 mM, respectively. The final concentration of SMP protein was 30 μg/ml. Values are means ± S.E. (*n* = 3).

SMPs were fused with varying amounts of liposomes composed of phospholipids alone. The NADH oxidation in the fused SMPs was measured in the presence of AOX (0.10 mg AOX/mg SMP protein) and cyanide (4.0 mM). The NADH oxidation activity decreased proportionally to the degree of enrichment of the SMP bilayer with liposomal phospholipids (*closed circles* in Fig. 3). When SMPs were fused with liposomes containing 11.8 (±2.7) mM UQ_10_ (Fig. S3*D*), the NADH oxidation activity slightly increased in a low concentration range of the fused liposomes, but then began to decline with a further increase in the incorporated liposomal phospholipids (*open circles* in Fig. 3). In all ranges of the incorporated phospholipids, the NADH oxidation activities in SMPs containing exogenous UQ_10_ were higher than those containing no exogenous UQ. These results well-match those reported by Schneider *et al*. (40), although they measured the NADH oxidase activity using rat liver mitoplasts (Fig. S3*B*). Thus, SMPs were successfully fused with the liposomes composed of phospholipids alone or phospholipids plus UQ_10_.

The ability of exogenous UQ_10_ to restore the electron transfer activity decreased after the phospholipid incorporation, supporting the idea that UQ_10_ diffuses independently and individually in the lateral plane of the inner mitochondrial membrane during its catalytic cycle (40, 43). This was also corroborated by a recent study using bovine heart SMPs supplemented with AOX in the presence of cyanide, in which endogenous UQ_10_/UQ_10_H_2_ freely mediated electron transfer between complex I and AOX (Fig. S3*C*), indicating that the proposed UQ_10_/UQ_10_H_2_ channeling inside the respiratory supercomplexes is not required to support respiration (41).

### The electron transfer activities of OS-UQs in the SMPs-Lipo-AOX system

**Figure 4:**
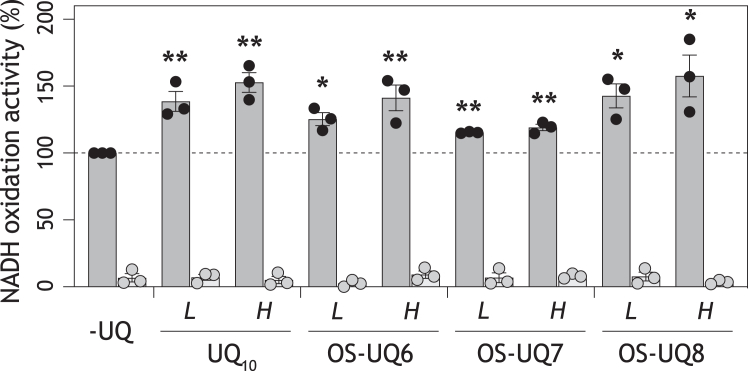
**The NADH oxidation mediated by OS-UQs in the SMPs-Lipo-AOX system.** The NADH oxidation rates determined in the SMPs-Lipo-AOX system fused with the liposomes (2.0 mg phospholipids/mg SMP protein) incorporating UQ_10_ (11.8 (±2.7) and 18.4 (±4.8) mM), OS-UQ6 (10.4 (±1.5) and 13.8 (±0.8) mM), OS-UQ7 (17.4 (±1.5) and 22.6 (±3.6) mM), or OS-UQ8 (8.9 (±1.2) and 15.3 (±1.2) mM) are shown. “*L*” and “*H*” present low and high concentrations of each UQ, respectively. The NADH oxidation rate measured in the system incorporating no exogenous UQ (“*-UQ*”) is presented as a control (100%, 0.23 (±0.05) μmol NADH/min/mg SMP protein). The final SMP protein concentration was set to 30 μg/ml. The electron transfer was almost completely inhibited by 1.0 μM ascofuranone (*gray data points*). ∗*p* < 0.05, ∗∗*p*< 0.01 compared with the control (one-way ANOVA followed by Dunnett’s test).

We prepared the SMPs fused with liposomes (2.0 mg phospholipids/mg SMP protein) containing no or a chosen UQ and measured NADH oxidation in the presence of AOX and cyanide (*i.e.*, in the SMPs-Lipo-AOX system, Fig. S3*D*). The NADH oxidation rate measured in the SMPs-Lipo-AOX incorporating no exogenous UQ is presented as a control (100%) in Figure 4 (marked “*-UQ*”). In this case, NADH oxidation proceeds via endogenous UQ_10_-mediated electron transfer between complex I and AOX (41). The electron transfer was almost completely inhibited by bullatacin (an inhibitor of complex I) or ascofuranone (an inhibitor of AOX); the latter is shown in Figure 4.

When the SMPs fused with the liposomes, which were composed of the same amount of total phospholipids and incorporated 11.8 (±2.7) or 18.4 (±4.8) mM of UQ_10_, were tested, the rates of the steady state NADH oxidation increased by ∼130 and ∼150% of the control, respectively (Fig. 4). We note that the experiment using the SMPs fused with liposomes incorporating higher concentrations of UQ_10_ (>∼20 mM) was not feasible because we were unable to prepare such liposomes due to enormously high pressure during the extrusion operation, which we had not experienced in the preparation of the liposomes incorporating lower concentrations of UQ_10_. This experimental restriction was also the case for other hydrophobic UQs.

Notably, the NADH oxidation rates determined in the SMPs-Lipo-AOX system fused with liposomes containing OS-UQ6 (10.4 (±1.5) and 13.8 (±0.8) mM), OS-UQ7 (17.4 (±1.5) and 22.6 (±3.6) mM), or OS-UQ8 (8.9 (±1.2) and 15.3 (±1.2) mM) were greater than the control and comparable to those containing UQ_10_ (Fig. 4). NADH oxidation by these OS-UQs was completely inhibited by bullatacin or ascofuranone (Fig. 4). For each OS-UQ, the NADH oxidation rates in the system incorporating a higher concentration of OS-UQ tended to be greater than that of a lower concentration, although there was no statistically significant difference between the two based on a Tukey test (>95%). Overall, the results reveal that OS-UQ6−OS-UQ8 can be catalyzed by the native complex I and mediate the electron transfer between complex I and AOX. Based on the crystallographic structure of AOX (42, 44), the predicted ubiquinol oxidation site nearby a diiron active center is accessed from membrane interior via a short cavity: two (or three) isoprene units of the ubiquinol molecule may be accommodated in the cavity. Therefore, the bulky block of OS-UQ6−OS-UQ8 may be outside the cavity of AOX and not limit the access of the quinol ring to the oxidation site.

We previously demonstrated that amphiphilic OS-UQ2 and OS-UQ3 can be catalytically reduced by the native complex I in ordinary SMPs (29). It may be interesting to elucidate whether this is also the case for the native complex I in the current SMPs-Lipo-AOX system. However, measurement of their electron transfer between complex I and AOX was not feasible because reduced OS-UQ2 and OS-UQ3 cannot function as substrates of AOX (29), as described in the Introduction section. Alternatively, these OS-UQs were directly added to the SMPs-Lipo-AOX incorporating no exogenous UQ, to give a final concentration of 20 μM in the reaction medium, in the presence of NADH (100 μM) and cyanide, and the initial NADH oxidation rate was determined, as conduct in the usual NADH-UQ oxidoreduction assay using SMPs. The vehicle-treated control NADH oxidation was attributable to the endogenous UQ_10_-mediated electron transfer between complex I and AOX (Fig. S4). The NADH oxidation rates after the addition of OS-UQ2 and OS-UQ3 were clearly enhanced compared with the control (Fig. S4). The rates of OS-UQ2 and UQ_1_ were higher than those of more hydrophobic analogues OS-UQ3 and UQ_2_, respectively, as observed with ordinary SMPs (27). This is because water solubilities of the former are higher than those of the latter. The NADH oxidation by these UQs was completely inhibited by bullatacin. Altogether, the results reveal that OS-UQ2 and OS-UQ3 also serve as substrates of the native complex I in the SMPs-Lipo-AOX system.

### The proton translocation coupled with the reduction of OS-UQs in the SMPs-Lipo-AOX

**Figure 5:**
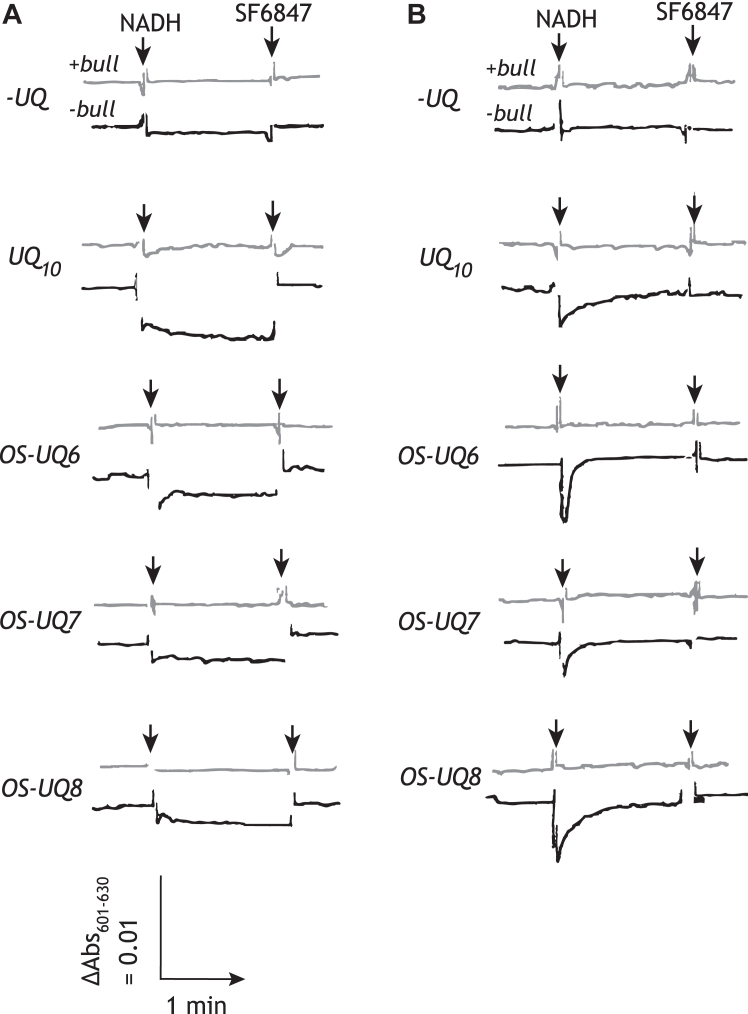
**The proton translocation coupled with the reduction of OS-UQs in the fused SMPs**. The membrane potential formation in the SMPs-Lipo-AOX (*Panel A*) or SMPs-Lipo without AOX (*Panel B*) system was monitored by following absorbance changes of oxonol VI (601–630 nm). The concentrations of UQ in the liposomes were as follows: UQ_10_; 18.4 (±4.8) mM, OS-UQ6; 13.8 (±0.8) mM, OS-UQ7; 22.6 (±3.6) mM, and OS-UQ8; 15.3 (±1.2) mM. The *gray* and *black traces* represent absorbance in the presence (“*+bull*”) or absence (“*-bull*”) of bullatacin (0.10 μM), respectively. No membrane potential was formed in the presence of 0.10 μM bullatacin in all cases. The *arrows* indicate the addition of NADH (100 μM) or SF6847 (0.10 μM). The SMP protein concentration was set to 90 μg/ml. The traces are representative of three experiments from 2 to 3 independent SMPs-Lipo preparations.

To assess whether the reduction of OS-UQ6−OS-UQ8 is coupled to proton translocation, we examined membrane potential formation in the SMPs-Lipo-AOX system by monitoring changes in the absorbance of oxonol VI. A steady formation of the membrane potential, which was dissipated by the addition of uncoupler SF6847, was determined for UQ_10_ (as a control) and OS-UQ6−OS-UQ8 (Fig. 5*A*). No membrane potential was generated in the presence of 0.10 μM bullatacin. The membrane potential formation in the SMPs-Lipo-AOX containing no exogenous UQ (a trace “*-UQ*”) was smaller compared with that of these SMPs-Lipo-AOX. The results indicate that the electron transfer of OS-UQ6−OS-UQ8 is coupled with proton translocation.

Incidentally, the extent of the formed membrane potential was considered to be substantially small. For reference, using the same concentration of SMP protein, the membrane potential generated by the UQ_10_ reduction in the SMPs-Lipo-AOX and that generated by directly adding UQ_2_ (20 μM) to ordinary SMPs were compared in Fig. S5. The low membrane potential in the SMPs-Lipo-AOX may be attributable to disturbance of the membrane integrity of SMPs due to the addition of DDM used for solubilizing AOX (41) and the fusion with liposomes (40).

On considering the above results, we also examined the membrane potential formation in SMPs fused with liposomes but without AOX. In this system, NADH oxidation completes in a short time because a limited amount of OS-UQ incorporated by the fusion is rapidly consumed by complex I and not recycled. As shown in Figure 5*B*, small but clear transient absorbance changes of oxonol VI were observed for the fused SMPs incorporating OS-UQs or UQ_10_ (as a control), but not in the presence of bullatacin. The absorbance change was negligibly small for the SMPs fused with the liposomes containing no UQ (a trace “*-UQ*”). These results also indicate that the reduction of OS-UQ6−OS-UQ8 by complex I is coupled to proton translocation. Taken together, it is noteworthy that although OS-UQ6 and OS-UQ7 were not catalytically reduced by the isolated complex I in the proteoliposomes (29), they can be catalyzed by the native enzyme in the current SMPs-Lipo-AOX system.

### The reactivity of PC-UQ4 in the complex I-proteoliposomes

To investigate the accessibility of the UQ head-ring of artificial UQs to the reaction site in complex I, we also previously produced phosphatidylcholine-UQ hybrid analogs (PC-UQ1−PC-UQ4, Figs. 1 and S2), which have an oleoyl group and UQ head-ring at the *sn*-1 and *sn*-2 glycerol positions, respectively (27). Given the phospholipid-like properties, it is difficult to image that the UQ head-ring of these hybrid compounds enters the canonical tunnel via the narrow entry point and reaches the deep reaction site near the cluster N2. Not as expected, PC-UQs were catalytically reduced by the native complex I in bovine SMPs. The reduction of PC-UQs and the proton-translocation coupled with the reduction were fully inhibitor-sensitive (27), indicating that their UQ head-ring can reach the physiological catalytic site. However, as the complex I-proteoliposomal system (Fig. S3*A*) was not available in the previous work (27), it remains to be clarified whether PC-UQs can be catalyzed by the isolated complex I. So, here we investigated the reaction of PC-UQs in the proteoliposomes using PC-UQ4 as a test compound.

**Figure 6:**
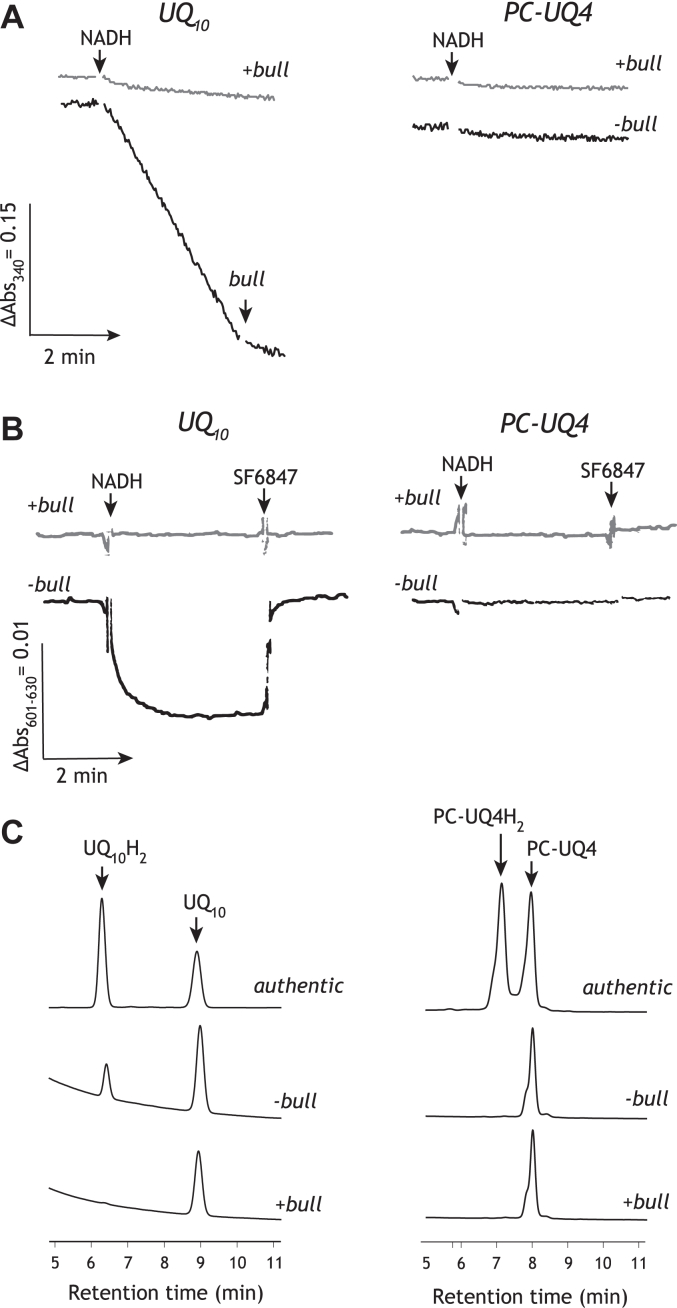
**The reactivity of PC-UQ4 in the complex I-proteoliposomes.***Panel A*, The NADH oxidation due to the UQ_10_- or PC-UQ4-mediated electron transfer between complex I and AOX was determined in the absence (*black lines*) or presence (*gray lines*) of 100 nM bullatacin. The proteoliposomes containing 3.3 (±0.6) mM UQ_10_ (3.3 (±0.1) μg complex I/mg phospholipids) or 6.9 (±1.0) mM PC-UQ4 (2.3 (±0.2) μg complex I/mg phospholipids) were prepared. The final complex I concentration was set to 1.8 μg/ml. *Panel B*, The membrane potential formation in the complex I-proteoliposomes was monitored by following absorbance changes of oxonol VI (601–630 nm) in the absence (*black lines*) or presence (*gray lines*) of 100 nM bullatacin. The complex I-proteoliposomes were the same samples as those used in the panel A. The final complex I concentration was set to 1.8 (±0.2) μg/ml. *Panel C*, The reduction of PC-UQ4 was monitored by determining its reduced form in the liposomal membrane by reverse-phase HPLC 5 min after the addition of NADH (1.2 mM). The upper chromatogram in each panel represents the retention times of the authentic oxidized and reduced forms of UQ tested. The middle and lower chromatograms represent the analytical results in the absence or presence of bullatacin (5.0 μM), respectively. The final complex I concentrations were 2.5 (±0.1) and 1.8 (±0.3) μg/ml for PC-UQ4 and UQ_10_, respectively. Data are representative of two experiments from two independent proteoliposome preparations.

We prepared, as a control, proteoliposomes containing 3.3 (±0.6) mM UQ_10_, and the steady NADH oxidation due to the UQ_10_-mediated electron transfer between complex I and AOX was determined (Fig. 6*A*). The electron transfer was completely blocked by the addition of bullatacin. The coupling of proton translocation to the reduction of UQ_10_ was confirmed by the formation of a membrane potential across the liposomal membrane, which was dissipated by addition of uncoupler SF6847 (Fig. 6*B*). The membrane potential was not formed in the presence of bullatacin. Notably, the electron transfer activity was not observed for the proteoliposomes containing 6.9 (±1.0) mM PC-UQ4 (Fig. 6*A*). Obviously, no membrane potential was generated in this case (Fig. 6*B*). These results indicate that PC-UQ4 does not function as an electron mediator between complex I and AOX. It is, however, unclear solely from these results which enzyme (complex I or AOX (or both)) could not recognize PC-UQ4 as a UQ substrate.

Therefore, we adopted another approach to examining the electron transfer ability of PC-UQ4; namely, the redox state of PC-UQ4, which was incorporated into the complex I-proteoliposomes without AOX, was assessed by reverse-phase HPLC analysis, as conducted in the previous work (29). If PC-UQ4 is catalytically reduced by the reconstituted complex I, its reduced form should accumulate in the liposomal membrane. However, no reduced form of PC-UQ4 was determined 5 min after the addition of 1.2 mM NADH (Fig. 6*C*). We confirmed, as a control, that a reduced form of UQ_10_ is produced under the same experimental conditions, but not in the presence of bullatacin (Fig. 6*C*). Taken together, we conclude that PC-UQ4 was catalytically reduced by the native complex I in SMPs (27) but not by the isolated enzyme reconstituted into the liposomes.

## Discussion

Structural studies have identified the long and narrow UQ-accessing tunnel within complex I from different biological sources (5, 6, 7, 8, 23, 45). To examine the physiological relevance of this tunnel model in a conceptionally straightforward way, we previously investigated whether a series of OS-UQs, which are too large to enter and transit the tunnel (Figs. 1 and S2), can be catalytically reduced by complex I using the native enzyme in the mitochondrial membrane and isolated enzyme reconstituted into liposomes (27). If the UQ reaction cavity is truly the narrow tunnel, the UQ head-ring of OS-UQs would not be able to reach the catalytic site nearby the cluster N2 unless the side chain length is longer than that of the predicted tunnel. In support of this, all OS-UQs, except OS-UQ8 that has a side chain longer than the tunnel (Fig. 1), were unable to be reduced by the isolated complex I in the proteoliposomes (27). However, amphiphilic OS-UQ2 and OS-UQ3 could be catalytically reduced by the native complex I in SMPs (27). This finding is difficult to reconcile with the canonical tunnel model. Thus, as contradictory results for the reactions of OS-UQs were obtained between the native and isolated complex I, the physiological relevance of the tunnel model has been left unsolved.

To explain the contradiction, we previously hypothesized that the UQ reaction cavity is flexibly “open” in the native complex I to allow OS-UQs to access the catalytic site, but the cavity is somewhat altered in the isolated enzyme by detergent-solubilizing from the mitochondrial membrane (29, 32). In fact, a cryo-EM study on mouse complex I with two piericidin A molecules bound suggested destabilization of the distal section of the tunnel structure due to detergent-solubilization (10). Nevertheless, a lack of information on the catalytic reaction of extremely hydrophobic OS-UQ6−OS-UQ8 by the native complex I in the mitochondrial membrane has prevented the assessment of the physiological relevance of the tunnel model. Therefore, here we provided the SMPs-Lipo-AOX assay system and uniformly examined the electron transfer activities of OS-UQs using the native enzyme. In this system, all OS-UQs tested were able to be reduced by the native enzyme, and the reduction was coupled with proton translocation. Both the electron transfer and proton translocation were completely inhibited by bullatacin. Thus, the reaction behaviors of OS-UQs are different between the native and isolated complex I. As the reason for this, it is most likely that there are some structural differences in the UQ reaction cavity between the two enzymes and, hence, steric obstructions that restrict the access of OS-UQs to the deep reaction site are also different, corroborating the previous hypothesis (29). Such different steric obstructions may also influence the reaction behaviors of artificial UQs that are less bulky than OS-UQs (32). It is noteworthy that the inactivity of OS-UQs in the complex I-proteoliposomes, except OS-UQ8 (27), can be no longer considered as evidence supporting the canonical tunnel model.

Even considering the UQ reaction cavity to be flexibly open in the native complex I, it is uncertain whether all OS-UQs reach the deep reaction site by passing through the main tunnel or if there is an alternative route(s). Based on chemical biology studies (27, 28, 29, 30, 31, 32), we previously proposed that the matrix-side interface region of flexible loops of the 49-kDa (β1–β2), ND1 (TMHs 5–6), and ND3 (TMHs 1–2) subunits may be a candidate for the alternative route(s) to the reaction site. Recent cryo-EM studies on short-chain UQ-bound porcine complex I (15) and medicinal biguanides-bound bovine complex I (17) may support this idea. Namely, Gu *et al*. (15) proposed that small short-chain UQs, such as UQ_1_, may diffuse into the reaction site through the matrix-side interface region. Bridges *et al*. (17) suggested that the UQ-accessing tunnel may become exposed to the matrix side in the deactive state of the enzyme, providing an alternative route for biguanides to enter the tunnel. Furthermore, a molecular dynamics simulation study reported that small UQ_1_ can leave from the UQ reaction cavity near the cluster N2 to the matrix-side medium through a route that is close to the TMHs 1 to 2 loop of ND3 (46). Thus, the likelihood of the existence of an alternative UQ-accessing route(s) in the enzyme cannot be fully ruled out, although there is still insufficient information on the route(s).

Regardless of the access routes, the UQ head-ring of OS-UQs must reach the deep reaction position to be reduced via cluster N2; therefore, it is almost inevitable that OS-UQs will encounter steric restraints during the transition. In such a situation, OS-UQ6 and OS-UQ7 may encounter more intense restraints compared with OS-UQ8, which can circumvent the restraints due to the longer isoprenyl tail. The predicted restraints may become more intense in the isolated complex I than in the native enzyme since the UQ reaction cavity may be altered in the former. For these reasons, the electron transfer activity of OS-UQ6 and OS-UQ7 may not have been determined with the isolated enzyme in the proteoliposomes (29). On the other hand, the steric restrictions that OS-UQ2 and OS-UQ3 encounter may be more intense compared with OS-UQ6–OS-UQ8 because the distances between the UQ head-ring and bulky block in the former are much shorter than in the latter. In light of this, we cannot exclude the possibility that amphiphilic OS-UQ2 and OS-UQ3 enter and pass through a different route from the hydrophobic OS-UQ6–OS-UQ8. If so, the most likely route may be the matrix-side interface region discussed above.

Given the phospholipid-like properties of PC-UQs (Fig. S2), their polar choline-phosphate head group may be arrayed in the membrane surface of the SMPs and proteoliposomes; therefore, it is difficult to image that their UQ head-ring enters the canonical tunnel and reaches the deep reaction site near the cluster 2. However, PC-UQ1–PC-UQ4 were catalyzed by the native complex I in ordinary SMPs in an inhibitor-sensitive manner (27). Contrary to this, here we revealed that PC-UQ4 cannot be catalyzed by the isolated enzyme reconstituted into liposomes. These different reaction behaviors would also be attributable to the structural differences in the UQ reaction cavity between the native and isolated complex I. While the chemical frameworks are markedly different between PC-UQs and OS-UQs, this does not necessarily mean that their UQ head-rings access the reaction site via entirely different routes. Considering that the polar UQ head-ring attached to the flexible acyl chain of PC-UQs can orient toward the membrane surface, the predicted entry of their UQ ring would also be located in the matrix-side surface region described earlier.

Mitochondrial complex I has been considered a promising target of anticancer reagents for glycolysis-deficient tumors (47, 48, 49, 50), though the risk associated with the clinical development of complex I inhibitors was recently pointed out (51). An accurate understanding of the mode of action of varying complex I inhibitors is essential for progress in anticancer reagent research as well as basic bioenergetic studies. Cryo-EM studies demonstrated that rotenone and piericidin A, which have largely different chemical frameworks, occupy the UQ-accessing tunnel in common (10, 11). Chung *et al*. (14) proposed that IACS-2858 (and IACS-010759 (47)), mubritinib (48), carboxyamidotriazole (49), and S1QELs (52) are able to adopt a similar “active” conformation near the entry of the tunnel. These structural studies not only mean that the inhibitors interfere with the UQ reaction in a competitive manner against substrate UQ but also that different inhibitors compete with each other for binding to the tunnel’s interior. However, our and other previous biochemical data obtained using the mitochondrial membrane samples are difficult to reconcile with these scenarios, examples follow.

1. Contrary to the behavior expected for competitive inhibitors, the maximum inhibition of the electron transfer by some S1QELs is saturated at incomplete levels (∼30−80%) (28, 29, 52).
2. S1QEL1.1 and S1QEL2.1 considerably reduce (by ∼2- to ∼10-fold) the binding affinities of rotenone and piericidin A to complex I at the concentration ranges exhibiting no electron transfer inhibition (53).
3. Excess piericidin A hardly suppresses the photoaffinity labeling of the ND1 subunit by a photoreactive quinone [^125^I]pUQ_*m*-1_ (Fig. S2), which can function as a substrate of both the native and isolated complex I (32), and by photoreactive IACS-010759 and S1QEL1.1 analogs (30).
4. A photoreactive amiloride-type inhibitor ([^125^I]PRA3) specifically labels the C-terminal region Thr227−Lys283 of the 39-kDa subunit (28), which does not comprise part of the canonical tunnel. However, this labeling is almost completely suppressed by excess rotenone and piericidin A (28).

Overall, the modes of action of a variety of complex I inhibitors cannot be described simply by the scenario that all inhibitor molecules occupy the canonical tunnel.

In conclusion, to elucidate the physiological relevance of the canonical UQ tunnel model, we investigated the native complex I-catalyzed reduction of OS-UQs using the SMPs-Lipo-AOX system. All OS-UQs tested could be catalyzed by the native enzyme, although they could not be catalyzed by the isolated enzyme reconstituted into liposomes, except for OS-UQ8 (29). The present study does not support the UQ tunnel model in the native complex I. The UQ reaction cavity may be flexibly open in the native enzyme to allow OS-UQs to access the reaction site. Additionally, the likelihood of the existence of an alternative UQ-accessing route(s) cannot be ruled out (*e.g.*, the matrix-side interface region of flexible loops of the 49-kDa, ND1, and ND3 subunits).

## Experimental procedures

### Materials

UQ_1_ and UQ_2_ were kind gifts from Eisai Co, Ltd (Tokyo, Japan). UQ_10_ was purchased from Sigma-Aldrich. OS-UQs and PC-UQ4 were the same samples as used previously (27, 29). All other reagents were of analytical grade.

### Preparation of bovine heart SMPs and measurements of electron transfer activity and membrane potential formation

SMPs were prepared from isolated bovine heart mitochondria according to the method of Matsuno-Yagi and Hatefi (54) and stored in sucrose-Tris buffer (0.25 M sucrose and 10 mM Tris/HCl, pH 7.4) at −80 °C. The NADH oxidase activity in SMPs was measured spectrophotometrically by following the oxidation of NADH with a Shimadzu UV-2600 instrument (340 nm, *ε* = 6.2 mM^-1^ cm^-1^) at 30 °C. The reaction medium (2.5 ml) contained 0.25 M sucrose, 1.0 mM MgCl_2_, and 50 mM phosphate buffer (pH 7.4). The final SMP protein concentration was 30 μg of protein/ml.

For the measurement of NADH oxidation via UQ-mediated electron transfer between complex I and AOX, the measurement was carried out using the same reaction medium in the presence of AOX (0.10 mg AOX/mg SMP protein) and KCN (4.0 mM). The final SMP protein concentration was 30 μg of protein/ml.

The membrane potential formation coupled with NADH oxidation was measured by following changes in the absorbance of oxonol VI (601–630 nm) with a Shimadzu UV-3000 instrument in the dual-wavelength mode in the reaction medium (2.5 ml) containing 0.25 M sucrose, 1.0 mM MgCl_2_, 0.8 μM antimycin A, 4.0 mM KCN, 2.5 μM oligomycin, 0.10 μM nigericin, 1.0 μM oxonol VI, and 50 mM phosphate buffer (pH 7.4) at 30 °C (27). The final mitochondrial protein concentration was 90 μg of protein/ml. The protonophoric uncoupler SF6847 was used at a final concentration of 0.10 μM to dissipate the membrane potential.

### Purification of complex I and AOX

Complex I was isolated from bovine heart mitochondria by solubilization with sodium deoxycholate and *n*-decyl-β-D-maltoside, and purified by sucrose density gradient centrifugation and anion-exchange column chromatography (55). The isolated enzyme was solubilized in a buffer (0.2% *n*-decyl-β-D-maltoside, 300 mM sucrose, 40 mM HEPES, pH 7.8) and stored at −80 °C.

The recombinant AOX from *T. brucei* was expressed in *Escherichia coli* (56). After the *E. coli* membrane was solubilized by *n*-octyl-β-D-glucoside, AOX was purified by cobalt-affinity chromatography (56). The isolated enzyme was solubilized in buffer (20 mM Tris-HCl, 200 mM imidazole, 0.042% (w/v) DDM, 50 mM MgSO_4_, 60 mM NaCl, 20% (v/v) glycerol, pH 7.3) and stored at −80 °C.

### Preparation of liposomes

Liposomes (large unilamellar vesicles) made of a phospholipid mixture extracted from the isolated bovine heart mitochondria, which predominantly contain phosphatidylcholine, phosphatidylethanolamine, and cardiolipin (29), were prepared by the extrusion method (57, 58). Stock solutions of the phospholipid mixture and chosen UQ in a methanol/chloroform (1:3, v/v) solution were mixed in the required proportions. A thin lipid film, which was obtained by evaporating organic solvents and was left under a vacuum overnight to remove residual organic solvents, was hydrated with Tris buffer (50 mM Tris-HCl, pH 7.4) or sucrose-Tris buffer (0.25 M sucrose and 10 mM Tris-HCl, pH 7.4) and vortexed. After seven rounds of freeze-thawing using liquid nitrogen and a 30−40 °C water bath under an N_2_ atmosphere, the lipid suspension was extruded through a 100-nm pore polycarbonate filter using a LiposoFast device (Avestin, Ottawa, Canada) to produce as many unilamella vesicles as possible.

The total phospholipid content in the liposomes was estimated using the phosphatidylcholine content, which was determined using the choline assay kit LabAssay Phospholipids (Wako Pure Chemicals, Osaka, Japan), and its ratio in the phospholipid mixture (∼40%, 29) on the assumption that each phospholipid was equally reconstituted into the liposomal membrane.

The UQ content in the liposomes was quantified by HPLC (Shimadzu LC-20AD system) using a standard calibration curve of the authentic sample (24). The suspension of proteoliposomes (10 μl) was mixed with a 10-fold volume of ethanol (90 μl), followed by sonication (1 min) and centrifugation (15,000 rpm, 5 min). The supernatant was separated on a reverse-phase column (COSMOSIL 5C_18_MS-II, 4.6 × 15 mm, Nacalai-Tesque, Kyoto, Japan) using a mixture of ethanol/methanol (3:2) containing 0.1% trifluoroacetic acid as a mobile phase with a flow rate of 0.8 ml/min. The elution profiles were monitored at 280 nm. The concentration of UQ in the liposomal membrane was estimated assuming that 1 mg of phospholipids occupies ∼1 μl; therefore, 1 nmol of UQ per mg phospholipids is equivalent to 1 mM (33).

### Fusion of SMPs with liposomes and measurements of electron transfer and membrane potential formation

SMPs were fused with liposomes essentially based on the procedures by Krämer (59). Briefly, bovine SMPs (1.0 mg protein in 15 μl sucrose-Tris buffer (0.25 M sucrose and 10 mM Tris/HCl, pH 7.4)) were gently mixed with the liposomes (2.0 mg phospholipids, 600 μl) prepared in the same buffer using a vortex mixer at room temperature. The liposomal membranes were composed of the phospholipid mixture alone or phospholipid mixture plus chosen UQ (∼5−20 mM). Then, the mixture of SMPs and liposomes was subjected to freeze-thawing using liquid nitrogen and a 30−40 °C water bath for seven rounds to accelerate membrane fusion. The fused SMP samples were kept on ice and used for the activity measurements on the same day. A portion of the fused SMPs was subjected to measurements of the electron transfer activity and membrane potential formation under the same experimental conditions as those of ordinary SMPs described above.

### Preparation of complex I-reconstituted proteoliposomes and determination of UQ content

The complex I-reconstituted proteoliposomes were prepared from liposomes (640 μl) containing the phospholipid mixture (10 mg) alone or the phospholipid mixture (10 mg) plus chosen UQ (10−20 mM). The liposomes were partially solubilized by the addition of 160 μl of *n*-octyl-D-glucoside in an aqueous 10% (w/v) solution. Then, the purified complex I (100 μg) was added to the solubilized liposomes (800 μl). The detergent was gradually removed from the mixture by the successive addition of SM Biobeads (Bio-Rad, 40 mg × 4 times) to afford the proteoliposomes. The complex I content in the proteoliposomes was determined by the amido black assay (60). Outward orientation of the reconstituted complex I, which was evaluated from acceleration of the electron transfer activity by the addition of the pore-forming antibiotic alamethicin (33), was ∼45% of the total enzyme.

The NADH oxidation activity via UQ-meditated electron transfer between complex I and AOX in the proteoliposomes and the membrane potential formation coupled with this NADH oxidation were determined by the same methods as those described for the assays using SMPs.

The UQ content in the proteoliposomes was quantified by HPLC analysis, as described above. For the analysis of PC-UQ4, the mobile phase comprised a linear gradient of solvent A (isopropanol/acetonitrile/1.0 M aqueous ammonium formate (600/390/10) containing 0.1% formic acid) and solvent B (isopropanol/acetonitrile/1.0 M aqueous ammonium formate (900/90/10) containing 0.1% formic acid). The gradient, starting at sample injection, was from 50 to 100% solvent B for 15 min.

To examine whether a reduced form of UQ was generated by complex I in the proteoliposomes, the proteoliposomes (2−5 μg of complex I/mg phospholipids) containing a single digit mM level of UQ were incubated with NADH (1.2 mM) in reaction medium (50 mM KCl and 10 mM Tris/SO_4_ (pH 7.5), 20 μl) at 30 °C on a heat block for 5 min. The NADH oxidation was stopped by adding argon-purged ethanol (80 μl), followed by gentle homogenization and centrifugation (16,000*g* at 4 °C for 5 min) (61). The supernatant was immediately analyzed by HPLC under the same experimental conditions as those for the measurement of the total UQ content in liposomes described above. Oxidized and reduced forms of each UQ were identified by retention times of the authentic samples. A reduced form of UQ was prepared by the method of Rieske (62). The elution profiles were monitored at 292.5 nm, an isosbestic point for oxidized and reduced forms.

## Data availability

All data described in the manuscript are contained within the manuscript and associated Supporting Information.

## Supporting information

This article contains supporting information (63).

## Conflict of interest

The authors declare that they have no conflicts of interest with the contents of this article.
