# Supplementary material for: Respiratory complex I in mitochondrial membrane catalyzes oversized ubiquinones
Source: J Biol Chem. 2023 Jun 30;299(8):105001. doi: 10.1016/j.jbc.2023.105001 (PMC10416054; doi:10.1016/j.jbc.2023.105001)
Supplement: Supplemental information [file mmc1.pdf]

SUPPORTING INFORMATION

Respiratory complex I in mitochondrial membrane catalyzes oversized ubiquinones

Ryo Ikunishi, Ryohei Otani, Takahiro Masuya, Kyoko Shinzawa-Itoh, Tomoo Shiba, Masatoshi Murai, and Hideto Miyoshi

Figure S1	p. S1
Figure S2	p. S2
Figure S3	p. S3
Figure S4	p. S4
Figure S5	p. S5

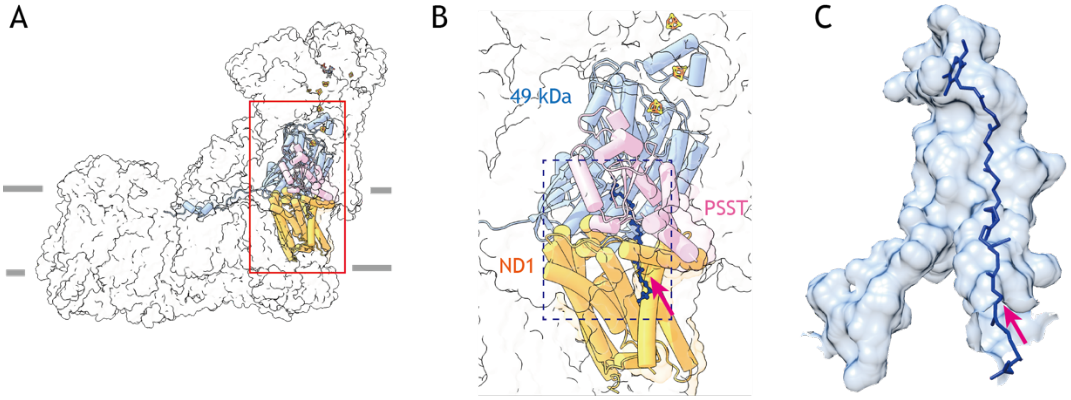

Figure S1

(A) Bovine complex I reconstituted into phospholipid nanodiscs (Protein data bank entry: 7QSK) using UCSF Chimera X 1.5 (ref. 63). (B) The binding model of exogenous UQ_{10} (*dark blue*) in the cavity formed by the 49-kDa, PSST, and ND1 subunits. (C) The tunnel-like cavity formed by the three subunits was generated by CASTp (ref. *63*) using a 1.4 Å probe and visualized by UCSF Chimera. The red arrows indicate the entrance of the cavity.

Reference:

63) Tian et al. (2018) *Nucleic Acids Res.* 46, W363-W367

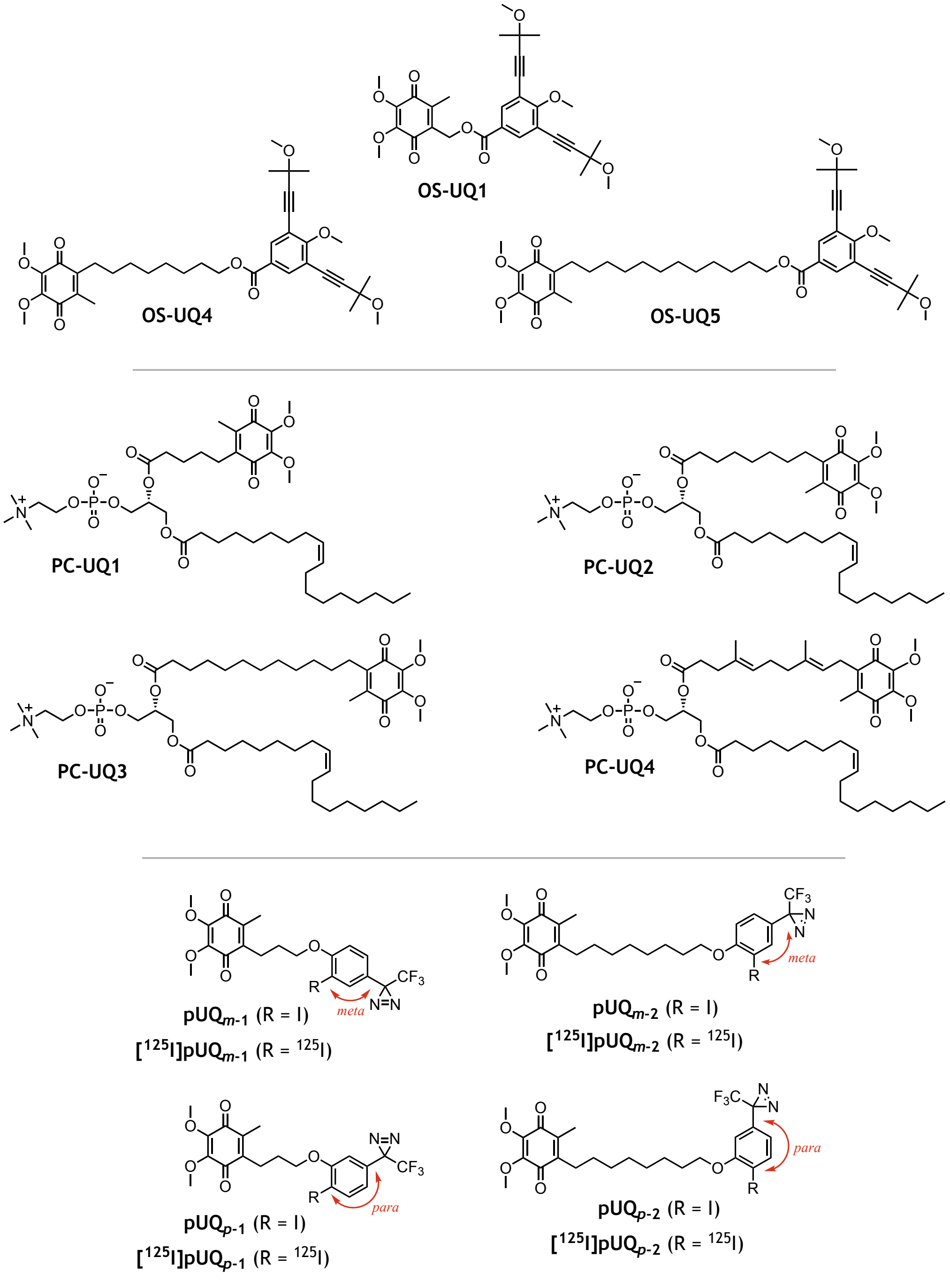

Figure S2

Structures of OS-UQs (ref. *29*), PC-UQs (ref. *27*), and pUQs (ref. *32*) are shown.

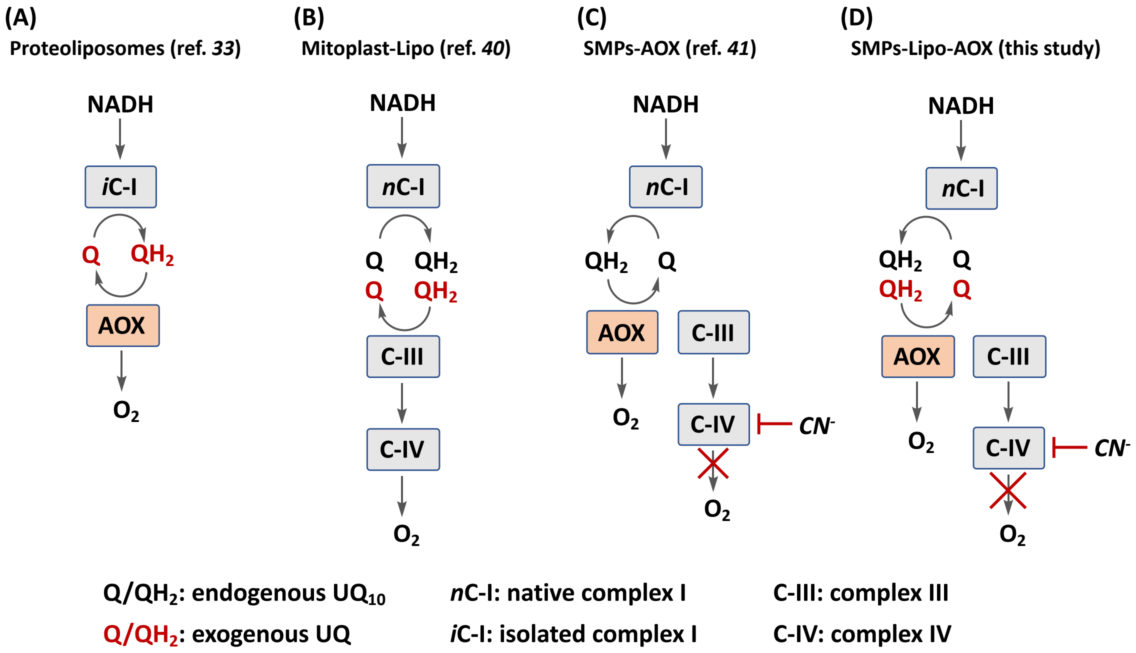

Figure S3

The electron flow (from NADH to O_2) in each assay system described in the text is schematically presented.

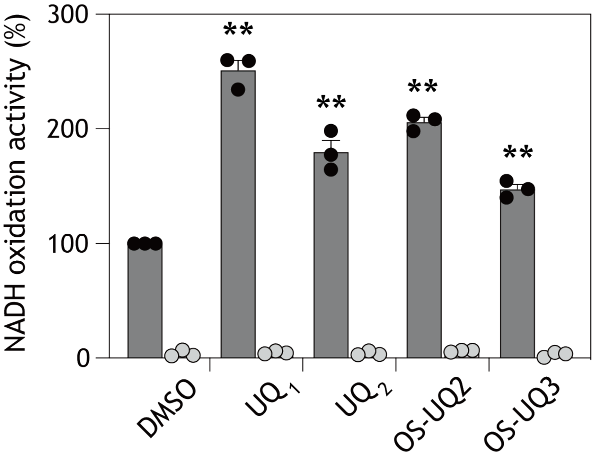

Figures S4

The NADH oxidation mediated by UQs in the SMPs-Lipo-AOX system. Each UQ was directly added to the SMPs-Lipo-AOX incorporating no exogenous UQ to give a final concentration of 20 μM in the presence of NADH (100 μM) and cyanide (4.0 mM). The initial NADH oxidation rates were determined. The electron transfer was almost completely inhibited by 0.1 μM bullatacin (*gray data points*). Values in graphs are means ± S.E. ($n = 3$). $^{**}P < 0.01$ compared with DMSO (one-way ANOVA followed by Dunnett's test).

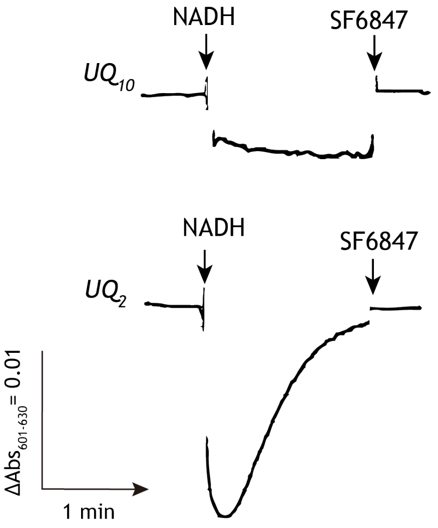

Figure S5

The membrane potential generated by UQ_{10} reduction in the SMPs-Lipo-AOX (*upper trace*) or directly adding UQ_2 (20 µM) to ordinary SMPs (*lower trace*) is shown. The NADH oxidation rates of the former and latter were 0.35 (± 0.04) and 0.22 (± 0.04) µmol NADH/min/mg SMP protein, respectively. The concentration of SMPs protein was set to 90 µg/ml in both assays. The upper trace for UQ_{10} is identical to that for UQ_{10} in Figure 5A.
